# A Fluorescent Biosensor for Sensitive Detection of *Salmonella* Typhimurium Using Low-Gradient Magnetic Field and Deep Learning via Faster Region-Based Convolutional Neural Network

**DOI:** 10.3390/bios11110447

**Published:** 2021-11-11

**Authors:** Qiwei Hu, Siyuan Wang, Hong Duan, Yuanjie Liu

**Affiliations:** 1 Key Laboratory of Agricultural Information Acquisition Technology, Ministry of Agriculture and Rural Affairs, China Agricultural University, Beijing 100083, China; s20193081423@cau.edu.cn (Q.H.); wangsiyuan@cau.edu.cn (S.W.); Duanhong@cau.edu.cn (H.D.); 2 Key Laboratory of Modern Precision Agriculture System Integration Research, Ministry of Education, China Agricultural University, Beijing 100083, China

**Keywords:** fluorescent biosensor, low-gradient magnetic field, deep learning, faster region-based convolutional neural networks, *Salmonella* detection

## Abstract

In this study, a fluorescent biosensor was developed for the sensitive detection of *Salmonella* typhimurium using a low-gradient magnetic field and deep learning via faster region-based convolutional neural networks (R-CNN) to recognize the fluorescent spots on the bacterial cells. First, magnetic nanobeads (MNBs) coated with capture antibodies were used to separate target bacteria from the sample background, resulting in the formation of magnetic bacteria. Then, fluorescein isothiocyanate fluorescent microspheres (FITC-FMs) modified with detection antibodies were used to label the magnetic bacteria, resulting in the formation of fluorescent bacteria. After the fluorescent bacteria were attracted against the bottom of an ELISA well using a low-gradient magnetic field, resulting in the conversion from a three-dimensional (spatial) distribution of the fluorescent bacteria to a two-dimensional (planar) distribution, the images of the fluorescent bacteria were finally collected using a high-resolution fluorescence microscope and processed using the faster R-CNN algorithm to calculate the number of the fluorescent spots for the determination of target bacteria. Under the optimal conditions, this biosensor was able to quantitatively detect *Salmonella* typhimurium from 6.9 × 10^1^ to 1.1 × 10^3^ CFU/mL within 2.5 h with the lower detection limit of 55 CFU/mL. The fluorescent biosensor has the potential to simultaneously detect multiple types of foodborne bacteria using MNBs coated with their capture antibodies and different fluorescent microspheres modified with their detection antibodies.

## 1. Introduction

In recent years, food safety has attracted more and more attention because it not only poses a threat to human health but also causes huge economic losses. According to the report of World Health Organization (WHO), it was estimated that about 600 million people became sick and 420 thousand people died due to the consumption of contaminated food. Furthermore, low-income and middle-income countries lose about 110 billion dollars due to unsafe food each year [1]. As a major foodborne pathogenic bacteria, *Salmonella* has been found in various foods and is responsible for millions of infections annually, such as fever, headache, nausea, vomiting, abdominal pain and diarrhea [2,3,4]. At present, traditional gold standard culture is still the most reliable method for the detection of foodborne pathogens; however, it often takes 2–4 days to obtain the final results [5,6]. Polymerase Chain Reaction (PCR) is a recommended method for the rapid and sensitive detection of foodborne bacteria; however, it requires a complex DNA extraction procedure [7,8]. Enzymatic Linked Immunosorbent Assay (ELISA) is another recommended method for the rapid detection of foodborne bacteria, but it often lacks sensitivity [9,10]. Thus, there is an urgent need to develop accurate, sensitive and rapid methods for the detection of foodborne pathogens.

Recently, fluorescent biosensors have received widespread attention due to their merits such as non-contact detection, easy operation, short time and high sensitivity [11,12,13,14]. Different fluorescent materials, such as fluorescein [15,16], quantum dots (QDs) [17,18] and fluorescent microspheres (FMs) [19], were often used to label target bacteria, resulting in the formation of the fluorescent bacteria in three-dimensional distributions. The intensity of the fluorescent signal is generally measured under excitation using a sensitive fluorescent meter to determine the concentration of target bacteria. An alternative to determine the concentration of target bacteria is to directly count the fluorescent bacteria [20,21,22]. A typical example was reported using a fluorescence microscope to directly count the fluorescent silica nanoparticles labeled as human chorionic gonadotropin and carcinoembryonic antigen [23]. Since a very high magnification was applied for counting the fluorescent targets due to the very small sizes of the targets, the selection of a representative zone was a big challenge. Furthermore, the three-dimensional distribution of the fluorescent bacteria in the aqueous solution could lead to the blocking and/or overlapping of the fluorescent signals, thus resulting in the underestimation of target bacteria. Therefore, the accuracy might be improved by converting the distribution of fluorescent bacteria from three-dimensional (spatial) to two-dimensional (planar). Many attempts have been made to address this problem (the spatial distribution of fluorescent signals). An artificial neural network was trained by Wu et al. [24] to refocus 2D fluorescent images of different focal planes onto a 3D surface using a software approach, which made up for the defects of fluorescence microscope image processing. For static image processing, an automated fluorescence microscope image analysis method was developed by Woźniak et al. [25] for the detection of *Pseudomonas aeruginosa*. First, the fluorescent images were preprocessed using image binarization, image sharpening and image contrast enhancement algorithms. Then, the shape features of the target bacteria were simplified and extracted using a traditional image processing method to identify the spherical and rod-shaped bacteria. Finally, statistical analysis was used to estimate the number of bacteria. This method was able to assist researchers and professionals in the rapid detection of signals from fluorescent microscope images; however, it has a complicated procedure due to the morphological variety of fluorescent spots and the limitations of traditional image analysis methods based on thresholds. Therefore, it is of great importance to develop simple, rapid and accurate methods to convert the spatial fluorescent signals into the planar fluorescent signals and identify the planar fluorescent signal.

In the past decade, more and more attention has been paid to deep learning. Compared to traditional machine learning, deep learning has shown its merits in microbiological analysis [26,27,28,29], such as strong learning ability, good adaptability and excellent performance. A typical example of deep learning was reported by Kanakasabapathy et al. [30] for the detection of embryos using inexpensive automated deep learning-based imaging systems, and more than 90% of embryos were successfully classified. Recently, the faster Region-based Convolutional Neural Network (R-CNN) [31] has been widely used for object recognition by first extracting candidate regions and then performing detailed classification. An interesting study was reported by Kutlu et al. [32] using faster R-CNN to simultaneously detect five types of white blood cells with a high accuracy of 95%. The faster R-CNN has shown its strong ability to identify the embryos or cells. In addition, Huang et al. [33] and Zieliński et al. [34] used a deep learning convolutional neural network to classify bacterial colony images, and their classification accuracies were 73% and 97.24%, respectively. However, their research work was based on specialized bacterial colony datasets [35] because bacteria are basically smaller and more difficult to be identified and classified.

**Scheme 1:**
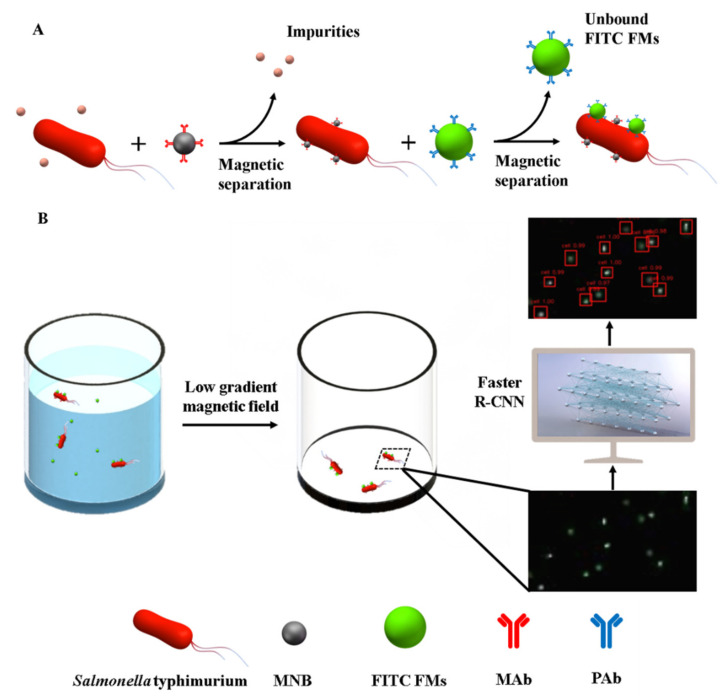
Schematic of this fluorescent biosensor. (**A**) Formation of fluorescent bacteria in the fluorescent biosensor. (**B**) Recognition and detection of fluorescent bacteria using a low-gradient magnetic field and deep learning via faster R-CNN.

In this study, the faster R-CNN deep learning algorithm was combined with fluorescent labeling to develop a sensitive biosensing method for the detection of *Salmonella* typhimurium. As shown as in Scheme 1, the magnetic nanobeads (MNBs) coated with the monoclonal antibodies (MAbs) against *Salmonella* typhimurium were first used for the specific separation of target bacteria from a sample background to form the magnetic bacteria. Then, the fluorescein isothiocyanate fluorescent microspheres (FITC FMs) modified with the polyclonal antibodies (PAbs) against *Salmonella* typhimurium were used for specific labeling of the magnetic bacteria to form the fluorescent bacteria. After the fluorescent bacteria were magnetically separated to remove the unbound FMs, they were concentrated in small volume of buffer solution and transferred into an ELISA well. Finally, the fluorescent bacteria were attracted against the bottom using a low-gradient magnetic field, resulting in a uniform planar distribution, and the fluorescent images were collected via scanning using an inverted fluorescence microscope and processed using the faster R-CNN deep learning algorithm to quantitatively determine the amount of target bacteria.

## 2. Materials and Methods

### 2.1. Materials

In this study, we used *Salmonella* typhimurium (ATCC 14028) as target pathogenic bacteria, and *Listeria monocytogenes* (ATCC 13932) and *E. coli* O157:H7 (ATCC 43888) as non-target pathogenic bacteria. We used the MAbs (1 mg/mL) from Meridian (Memphis, TN) and the PAbs (2.5 mg/mL) from Fitzgerald (Acton, MA, USA) to capture target bacteria specifically. The biotin labeling kit from Elabscience (Wuhan, China) was utilized to coat the MAbs with biotin. MNBs (150 nm, 1 mg/mL) modified with streptavidin from Ocean Nanotech (MHS-150-10, Dunedin, FL, USA) were utilized for immunomagnetic separation. N-(3-dimethylaminopropyl)-N’-ethylcarbodiimide hydrochloride (EDC·HCl) were used to activate the carboxyl groups on the surface of FITC FMs with the diameter of 500 nm (excitation wavelength: 465–495 nm; emission wavelength: 550 nm; concentration: 1 mg/mL) from Nanchang university. The cylindrical magnets from K&J Magnetics (Pipersville, PA, USA) were used to produce the low-gradient magnetic field. The holder for housing the magnets was printed using the Objet24 3D printer from Stratasys (Eden Prairie, MN, USA). Phosphate-buffered saline (PBS) from Sigma (St. Louis, MO, USA) was diluted 10 times with ultrapure water and it was used as a buffer solution. Tween-20 from Amresco (Solon, OH, USA) was used for cleaning. Bovine serum albumin (BSA) from Sigma Aldrich (New Jersey, USA) was used for the sealing of centrifugal tubes. The culture of bacteria was based on the brain heart infusion medium from Remel (Lenexa, KS, USA) and Luria–Bertani (LB) medium from Aoboxing Biotech (Beijing, China). All the solutions were prepared with ultrapure water, which was produced by Advantage A10 from Millipore (18.2 MΩ·cm, Billerica, MA, USA).

### 2.2. Preparation of the Bacteria

Prior to use, both target bacteria and non-target bacteria were stored with 50% glycerin at −20 °C. For culture, a 50 μL bacterial sample was added into 5 mL of LB liquid medium at 37 °C, with shaking at 180 rpm for 12–16 h. For enumeration, after the bacterial culture were diluted with sterile PBS to obtain different concentrations of bacterial samples, 50 μL bacteria samples were plated on the LB Agar plate and incubated at 37 °C for 16–22 h. Finally, the concentration of bacteria was calculated by counting the visible colonies.

### 2.3. Preparation of the Immune FITC FMs

First, 100 μg FITC FMs and 30 μg PAbs were added into 1.5 mL PB buffer solution (pH 6.0, 0.01 M). Then, 20 μg EDC·HCl was added to activate the carboxyl groups on the surface of the FMs. Finally, the immune FMs, with a final concentration of 0.1 mg/mL, were obtained after blocking with 150 μL BSA (10%) and centrifuging at 10,000 rpm for 15 min.

### 2.4. Development of the Low-Gradient Magnetic Field

The low-gradient magnetic field device was developed for converting spatial signals to planar signals. As Figure S1 shown in the supplementary materials, it mainly consisted of two parts: (1) a cylindrical magnet with the diameter of 30.5 mm and the height of 34 mm for the generation of a low-gradient magnetic field, and (2) a 3D-printed holder with the diameter of 32.5 mm and the height of 40 mm for housing of the magnet and the ELISA well with the diameter of 8.5 mm and the height of 6 mm at their accurate positions. Physical diagrams of the low-gradient magnetic field device are shown in Figure S1 in the supplementary materials. Hydration particle size analysis of FITC FMs and immune FITC FMs is shown in Figure S2 in the supplementary materials.

### 2.5. Acquisition of Fluorescent Images and Preparation of the DataSet

The acquisition of fluorescent images is the key to recognition and detection of target bacteria. For image acquisition, after spatial signals were converted to planar signals using the low-gradient magnetic field, the inverted fluorescence microscope was used at a magnification strength of 150 times to collect the fluorescent images via scanning. For *Salmonella* typhimurium at a concentration of 1.4 × 10^5^ CFU/mL, 10 fluorescent images (5000 × 3000 pixels) were first collected and preprocessed by OpenCV-Python image sharpening, brightness enhancement and contrast enhancement algorithms. Then, each fluorescent image was divided into 100 smaller ones (500 × 300 pixels), which were annotated using the LabelImg software and shown in Figure S3 in the supplementary materials. Finally, Python-based script files were used to produce the dataset, and the images of the fluorescent bacteria, the labeling information of the fluorescent images and the segmentation information of the dataset were organized into three different folders, respectively.

### 2.6. The Establishment of the Faster R-CNN Model

The establishment of the faster R-CNN model was central to the identification of *Salmonella* typhimurium. It mainly included the establishment of a graphics processing unit (GPU) environment, verification of the original model, and training of the model. For the establishment of the GPU environment, the compute unified device architecture (CUDA) package was first installed on the Linux operating system under the Huawei Cloud ModelArts development environment (GPU: 1 × p100, CPU: 8 core 64 GiB). Then, Torch, Torchvision, Scikit-image, Matplotlib and other necessary third-party libraries were installed via Linux command-line. For the verification of the original model, the feasibility of the original model was verified by a pre-trained model and a Pascal Visual Object Classes (VOC2007) dataset. The algorithm used is an open-source project on the GitHub website [36]. For the model training with the fluorescent image dataset, LabelImg [37] was first employed to manually label the fluorescent images according to the format of the VOC2007 dataset. Then, the pre-trained model was retrained to establish and fit the model based on the fluorescent image dataset. In every training epoch, the training set and validation set were used to adjust the inner weights and obtain the optimal model, respectively.

### 2.7. Separation, Identification and Detection of Target Bacteria

For the separation of the target bacteria, 20 μg immune MNBs were first added to different concentrations of target bacteria (1100 CFU/mL, 550 CFU/mL, 275 CFU/mL, 138 CFU/mL and 69 CFU/mL) and incubated for 45 min to form the magnetic bacteria (MNB-bacteria complexes). After washing twice with 500 μL PBST (PBS with 0.05% Tween-20) to remove the sample background, 4 μg immune FITC FMs were then added into the magnetic bacteria and incubated for 45 min to form the fluorescent bacteria (MNB-bacteria-FITC FM complexes). Finally, after washing twice with 500 μL PBST to remove the excessive FITC FMs, the fluorescent bacteria were concentrated in 200 μL PBS solution and attracted to the bottom of an ELISA well in the presence of the low-gradient magnetic field. For the identification and detection of the target bacteria, the inverted fluorescence microscope was used to collect 20 fluorescent images, which were used as the test set to identify the fluorescent bacteria for quantitative detection of target bacteria.

### 2.8. Detection of Target Bacteria in Spiked Milk

To verify the applicability of our proposed biosensor, we purchased fresh milk from a supermarket and prepared the spiked samples. Based on the Chinese national standards on the detection of pathogens (GB 4789.4-2016), 25 mL milk was first taken and sufficiently mixed with 225 mL sterile PBS . Then, different concentrations of *Salmonella* typhimurium (1100 CFU/mL, 550 CFU/mL, 275 CFU/mL, 138 CFU/mL and 69 CFU/mL) were added to the milk samples to obtain the spiked milk samples. Finally, the target bacteria in each spiked sample were first separated using the immune MNBs, then labelled with the immune FITC FMs, and finally detected using this biosensor.

## 3. Results and Discussion

### 3.1. Identification of Fluorescent Spots Using the Faster R-CNN Model

**Figure 1:**
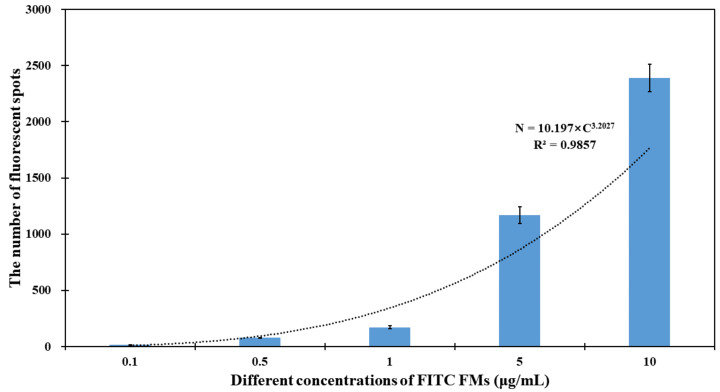
The relationship between the identification results and the concentration of FITC FMs.

To demonstrate the feasibility of the faster R-CNN model for the identification of fluorescent spots, the images for different concentrations of the FITC FMs ranging from 50 to 0.1 μg/mL were collected under the excitation of an ultraviolet lamp. As Figure S4 shown in the supplementary materials, higher concentrations of FITC FMs have an obviously higher density of fluorescent spots. A fluorescent image at the concentration of 50 μg/mL was first divided into 100 smaller images. Afterwards, they were annotated by LabelImg, and all the images were divided into 90 training images and 10 validation images. The whole dataset was traversed 10 times and the highest recognition precision of the model for the validation set was about 89.2%. Finally, the fluorescent images for different concentrations (10, 5, 1, 0.5 and 0.1 μg/mL) of FITC FMs were used as a test set. The identification results are plotted in Figure S4. As shown in Figure 1, the results showed that the average counting identification results of the faster R-CNN model for the test set were 2392, 1168, 171, 77 and 14, respectively, and the amount of fluorescent spots (***N***) had a linear relationship with the logarithm of the concentration of FITC FMs (***C***) and could be expressed as ***N*** = 10.197 × ***C*** ^3.2027^.

### 3.2. Transformation of the Fluorescent Bacteria from Spatial to Planar

**Figure 2:**
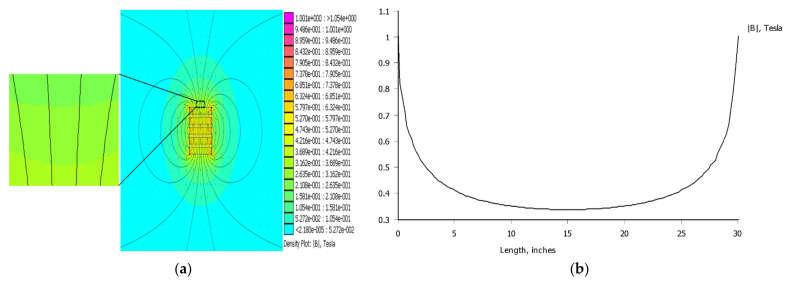

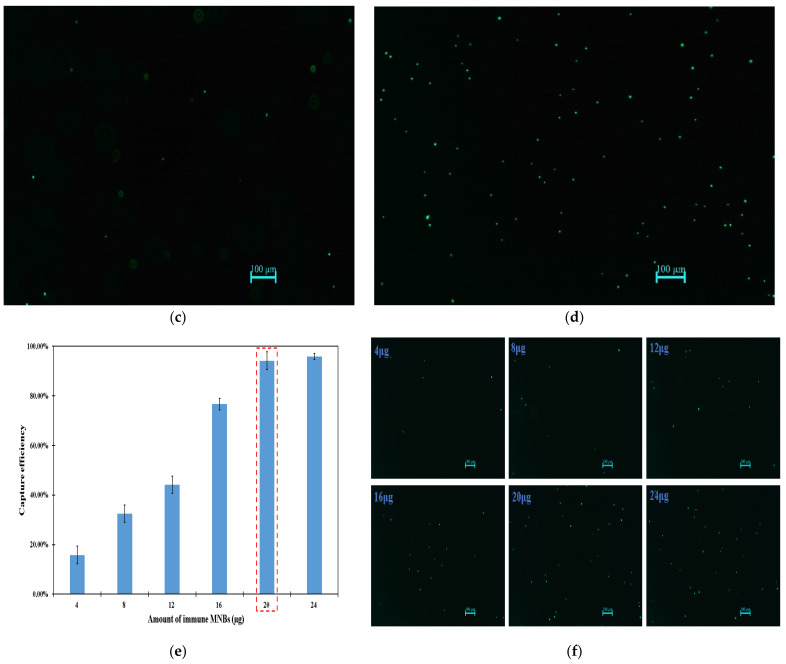
Characterization of low-gradient magnetic field and effect of MNBs on fluorescent signals. (**a**) Simulation of the magnetic field distribution on the surface of the cylindrical magnets. (**b**) Simulation of the magnetic field intensity on the surface of the cylindrical magnets. (**c**) The distribution of fluorescent spots without low-gradient magnetic field. (**d**) The distribution of fluorescent spots with low-gradient magnetic field. (**e**) Optimization of the amount of immune MNBs. (**f**) Fluorescence images for different dosages of MNBs. The scale bar is 100 μm.

The uniform distribution of the fluorescent bacteria on the bottom of ELISA well is the key to the accurate and sensitive detection of target bacteria. In general, the fluorescent bacteria were distributed in three-dimensional space and this inevitably resulted in the overlapping of the fluorescent bacteria from the view of the fluorescence microscope. Thus, the low-gradient magnetic field was developed to attract the fluorescent bacteria against the bottom of the ELISA well. To understand the distribution of the low-gradient magnetic field, the finite element magnetic simulation software (FEMM) was used for simulation of the magnetic field. As shown as in Figure 2a,b the magnetic field at the ELISA well had a mean magnetic intensity of 0.424 T and a mean gradient of 1.84 T/m.

The size of the fluorescent bacteria (spots) is an important criterion for the identification of the target bacteria. Thus, the fluorescent spots were collected before and after the low-gradient magnetic field was applied. As shown as in Figure 2c,d, the fluorescent spots before applying the magnetic field (mean diameter: 11.11 μm; standard deviation: 8.19 μm) were of a larger size than those after applying the magnetic field (mean diameter: 2.77 μm; standard deviation: 0.41 μm). This was because the fluorescent bacteria were distributed in the three-dimensional space before applying the magnetic field and they had bigger halos when some of them were not at the focal plane. However, after applying the magnetic field, they were attracted to the bottom and had the same focal plane. This verified that the spatial signals could be successfully transformed into the planar signals using this low-gradient magnetic field. The distribution of complexes with and without low-gradient magnetic fields are shown in Figure S1 in the supplementary materials.

Furthermore, in order to fully detect the fluorescent spots on the bottom of the ELISA container, the area of the bottom surface of the ELISA container and the number of collected images needed to be considered. The bottom area of the ELISA container can be calculated by the following expression:

S = π × R^2^ (1)

where π is PI, R is the inner radius of the ELISA container, S is the base area of the ELISA container. It was calculated that S is approximately 32.17 mm^2^. According to the image area calculation function of the inverted fluorescence microscope, the area of each field of view was approximately 1.3465 mm^2^ at the magnification of 150. When twenty fields of view were chosen, total detection area of each sample (about 26.93 mm^2^) exceeded 80% of the entire base area of the ELISA container. Furthermore, in order to verify the uniform distribution of fluorescent signals, three parallel experiments were conducted using target bacteria at the concentration of 1.4 × 10^3^ CFU/mL; detailed information is listed in Table S1. The results showed that the numbers of fluorescent spots in the nine scanned images were very close (average: 16; standard deviation: about 2). Therefore, several images could be collected by several fields of view for statistical calculation of the number of fluorescent spots for the detection of target bacteria.

In this study, the amount of immune MNBs had an impact on the strength of fluorescent signals. On the one hand, if the amount was too little to capture enough bacteria, the target bacteria could not be fully detected by this fluorescent biosensor and it would result in low sensitivity. On the other hand, if the amount was too much, MNBs not only absorbed the excitation light but also absorbed the fluorescence emitted by the fluorescent bacteria, which led to the suppression of the strength of the fluorescent signals. Therefore, different amounts of immune MNBs (4 μg, 8 μg, 12 μg, 16 μg, 20 μg and 24 μg) were used to capture target bacteria to calculate the capture efficiency using target bacteria at the concentration of 1.1 × 10^4^ CFU/mL. As shown in Figure 2e, when the amount of immune MNBs increased from 4 to 20 μg, the capture efficiency increased from 15.8 to 91.1%, but when the amount of immune MNBs continued to increase to 24 μg, the capture efficiency of bacteria no longer showed a clear increasing trend. Therefore, the optimal amount of immune MNBs used in this study was 20 μg.

### 3.3. Optimization of the Faster R-CNN Deep Learning Algorithm

At present, fluorescent images are often processed using classical methods based on threshold and deep learning algorithms using CNNs. To the best of our knowledge, the available methods for the enumeration of fluorescent points are almost all based on thresholds. However, it is difficult to set a threshold for the entire image. Thus, the threshold-based contour counting algorithm and faster R-CNN object detection algorithm were simultaneously used to compare their identification performance of the fluorescent spots.

**Figure 3:**
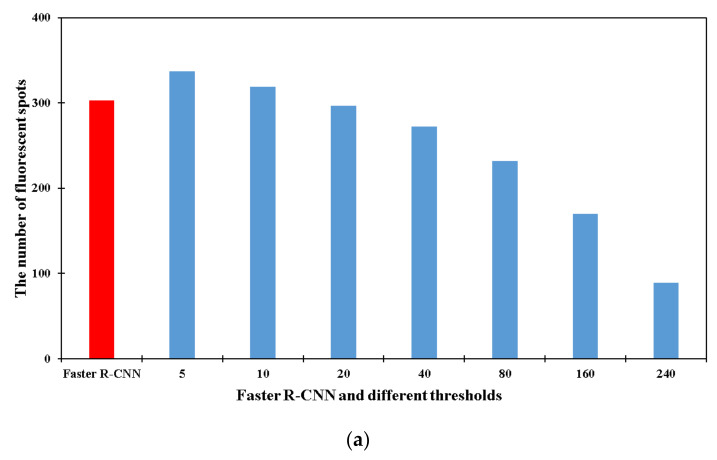

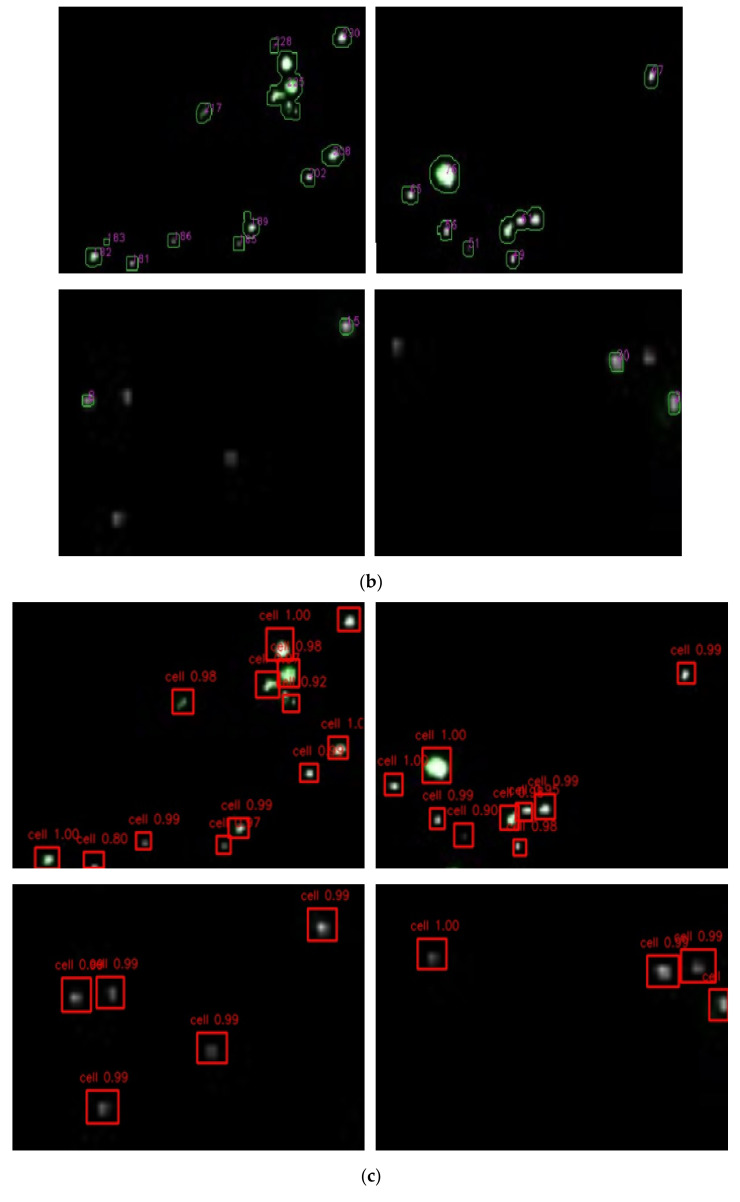

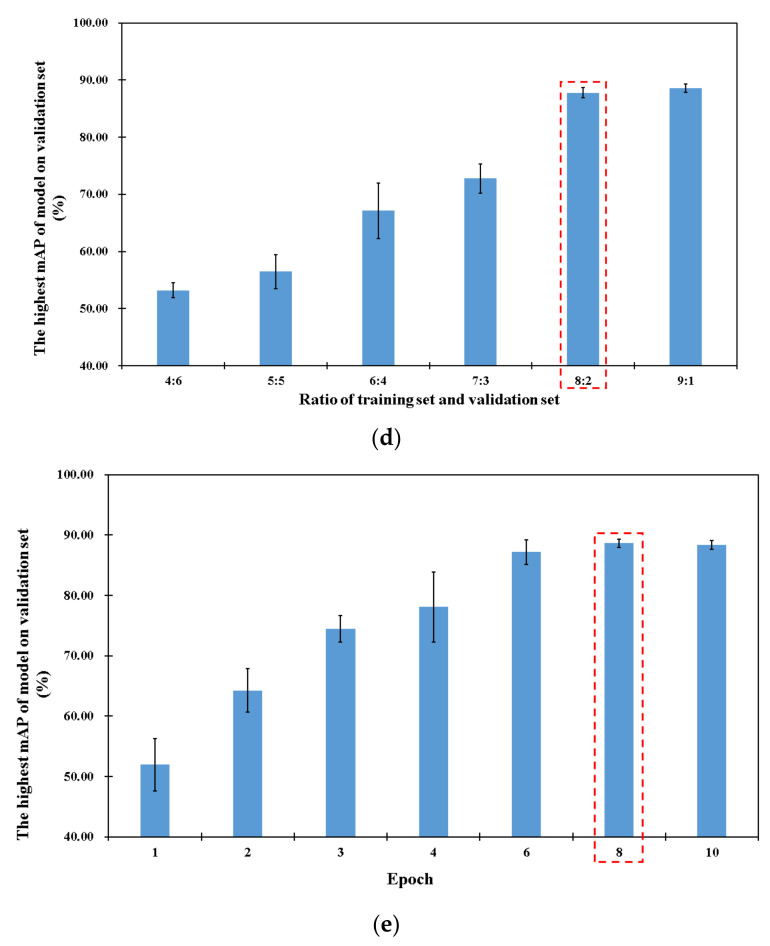
Comparison of faster R-CNN algorithm with contour counting method. (**a**) Comparison of the counting results between faster R-CNN based on deep learning and contour counting based on thresholds for fluorescent images at the concentration of 1.1 × 10^3^ CFU/mL. (**b**) The counting situation of contour counting method on fluorescent images. (**c**) The counting situation of faster R-CNN on fluorescent images. (**d**) Optimization of the ratio of the training set to the validation set. (**e**) Optimization of the training epoch.

For the contour counting algorithm, different thresholds from 5 to 240 were set to identify the fluorescent points. As shown in Figure 3a, when the threshold increased from 5 to 240, the number of fluorescent spots decreased from 337 to 89, which indicated that the result of contour counting algorithm based on threshold was unstable and not suitable for the identification of fluorescent spots. For the faster R-CNN algorithm, the result of the optimal model was 303. As shown in Figure 3b, the higher the threshold value, the less likely fluorescent spots with low brightness were to be identified; thus, some signals may be missed by the method. The lower the threshold value, the more likely some fluorescent spots—including background noise—would be identified as objects; thus, a high detection result could be obtained by a low threshold value. In contrast, as shown in Figure 3c, excellent recognition performance could be accomplished using our optimal model. This performance was based on a dataset of fluorescent images, and it was found that different fluorescent spots could be accurately identified in this way.

The above phenomena (different results for the same fluorescent images with the contour counting method and faster R-CNN) could be explained by following reasons: (1) The brightness levels on different fluorescent images were different and the brightness on different parts of one fluorescent image were different. Therefore, it was hard to use unified thresholds to fit all fluorescent images or different parts of one image. (2) Threshold methods were featured with less code, easy implementation and fast speed, but the threshold needed to be adjusted according to different images. Therefore, the results were unstable and the recognition accuracy was not high, because only shallow and local information of fluorescent images could be acquired by the threshold method. (3) Although the object detection algorithm was characterized by complex code, slow speed, and the need for a dataset of high quality as well as a complete training strategy, deep and global information of the whole dataset could be obtained. The model was fitted based on the whole dataset of fluorescent images. The optimal model had many convolution kennels, and they could extract low-level features such as brightness, colors and shapes and map them to high-level features; therefore, the natural features of fluorescent spots were learned.

Furthermore, fluorescent spots were found to have a tendency of aggregation in the process of the experiments. Aggregated fluorescent spots could not be identified by the contour counting method, while this problem could be solved by the faster R-CNN algorithm. In a word, the threshold method was not stable in recognition of fluorescent images; faster R-CNN was stable and it showed great advantages in object detection.

However, due to different characteristics of different datasets, the initial performance of the model on the dataset of fluorescent images was not very high, so hyper-parameters needed to be adjusted and optimized. In this study, two hyper-parameters were chosen as optimized objects, and they were the training epoch as well as the ratio of the training set and the validation set, respectively. As we know, an epoch is that the entire dataset was traversed once. Because the contents of different datasets may be different, it is necessary to traverse the dataset of fluorescent images many times to fully extract and understand the features. The ratio of the training set to the validation set is defined as the proportion of the number of training set images to validation set images. On the one hand, if the number of pictures in training set is small, the phenomenon of under-fitting (bad on the training set, bad on the test set) will appear because the model is unable to fully learn the features of the dataset. On the other hand, if the number of pictures in the training set is too large, the phenomenon of over-fitting (good on the training set, bad on the test set) would be also appear, which would result in the poor performance of the model in the test set. Furthermore, the AP (average precision) is the recognition accuracy of the model in a single category and the mAP (mean average precision) is the mean of the sum of the recognition accuracy of the model in all categories. In this study, the mAP was equal to the AP because we only had one annotated category (cell) in our dataset. The value of the mAP was between (bad) 0 and 1 (good), and it was used to evaluate the performance of the model.

First, the epoch value of 10 was used as default epoch and different ratios (4:6, 5:5, 6:4, 7:3, 8:2 and 9:1) of the training set to the validation set were chosen to optimize the performance of the model. As shown in Figure 3d, when the ratio was increased from 4:6 to 8:2, the highest mAP of the model on validation set increased from 53.18 to 87.79%, and when the ratio was still enhanced, the mAP no longer appeared to increase. These could be explained by the following reasons: (1) When the ratio of the training set to the validation set was low, there were not enough data for the model to learn and understand the information contained within the dataset; thus, it was hard for the model to learn sufficient information regarding fluorescent spots, and the recognition ability of the model on the test set was low. (2) When the ratio was high, sufficient information could be learned from the training set; thus, the model showed good performance on the validation set. Therefore, the optimal ratio of training set to validation set was set to 8:2.

For the further improvement of the recognition accuracy of the model on the validation set, the epoch was optimized using different epoch values (1, 2, 3, 4, 6, 8 and 10) based on the optimal ratio (8:2) of the training set to the validation set. As shown as in Figure 3e, when the epoch was increased from 1 to 8, the highest mAP of the model on the validation set improved from 51.97 to 88.62% and there was little change with the continuous increasing of the epoch from 8 to 10. This was probably due to the following reasons: (1) The characteristics of fluorescent spots were inherently simple, so a recognition accuracy of about 50% could be achieved through an epoch. (2) After preprocessing, the outline and morphology of the fluorescent spots were more obvious; therefore, natural information regarding the fluorescent spots could be obtained through the eight traversals of the training set. The mAP of the model might be improved by increasing the training epoch, but this could potentially lead to over-fitting. Therefore, the optimal epoch was found to be eight.

### 3.4. Detection of Target Bacteria Using This Fluorescent Biosensor

**Figure 4:**
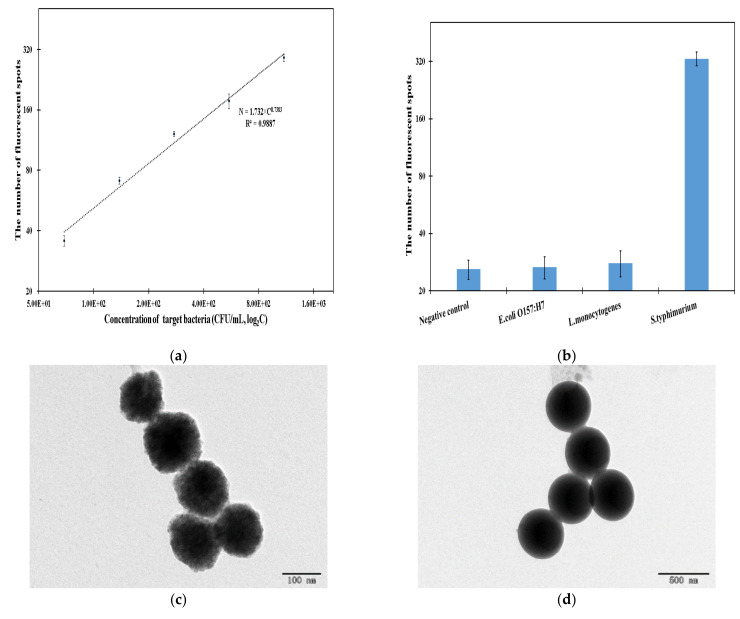

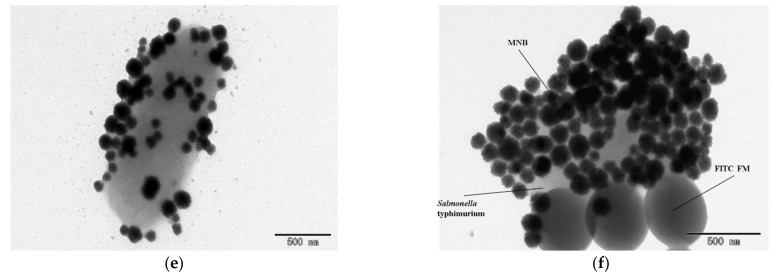
The performance characterization of this proposed fluorescent biosensor and the characterization of the materials used in this study. (**a**) The calibration curve of this fluorescent biosensor (*N* = 3). (**b**) The specificity of this biosensor (*N* = 3). (**c**) The TEM image of the MNBs. (**d**) The TEM image of the immune FITC FMs. (**e**) The TEM image of the magnetic bacteria. (**f**) The TEM image of the fluorescent bacteria.

To quantitatively detect unknown concentrations of *Salmonella* typhimurium, the calibration curve was established using this fluorescent biosensor to detect different concentrations of target bacteria from 69 to 1100 CFU/mL under the optimal conditions. As shown as in Figure 4a, the number of fluorescent spots (***N***) increased with the concentration of target bacteria (***C***), and thus, the calibration curve could be expressed as:

***N*** = 1.732 × ***C***^0.7383^ (2)

Based on the signal-to-noise ratio of three, the lower detection limit of this fluorescent biosensor was calculated as 55 CFU/mL. Compared to some recently reported fluorescent biosensors, this fluorescent biosensor showed higher or comparable sensitivity, shorter detection times and an easier detection procedure. The good performance of the biosensor was mainly due to following reasons: (1) The transformation of the fluorescent bacteria from spatial to planar using the low-gradient magnetic field, which resulted in a reduced halo effect of the fluorescent spots. (2) The fluorescent bacteria were automatically identified and counted using the faster R-CNN deep learning algorithm, which resulted in more accurate measurements of the fluorescent bacteria. (3) The specific separation and efficient enrichment of the target bacteria using the immunomagnetic separation, which resulted in less interference in the fluorescent detection.

Moreover, the specificity of this fluorescent biosensor was evaluated using *Salmonella* typhimurium, *Listeria monocytogenes* and *E. coli* O157:H7 at the same concentration of 10^3^ CFU/mL. As shown in Figure 4b, the number of fluorescent spots for the target bacteria was 320, which was much more than those observed for the non-target bacteria (28 for *Listeria monocytogenes* and 27 for *E. coli* O157:H7) and the negative controls (26). This showed that this fluorescent biosensor had a good specificity, which was mainly due to the specificity of the MAbs and PAbs against the target bacteria. Furthermore, the MNBs, FITC FMs, magnetic bacteria and fluorescent bacteria were characterized by transmission electron microscopy (TEM); the results are shown in Figure 4c–f. In addition, this proposed fluorescent biosensor was compared with recently published fluorescent biosensors, as shown in Table S2.

### 3.5. Detection of Target Bacteria in Milk Sample

To further evaluate the applicability of this fluorescent biosensor for the detection of *Salmonella* typhimurium in real samples, milk was purchased from a local supermarket and spiked with target bacteria to prepare the bacterial samples at the concentrations of 1100 CFU/mL, 550 CFU/mL, 275 CFU/mL, 138 CFU/mL and 69 CFU/mL. All the spiked samples and the negative control (PBS) were tested three times in parallel. The recovery was calculated as the ratio of the detected bacteria to the added bacteria. As shown in Table 1, the recovery of this biosensor for different concentrations of *Salmonella* typhimurium ranged from 85.31 to 110.48%, with the mean recovery of 102.74%, indicating that this biosensor could be applied for the detection of *Salmonella* typhimurium in milk. More importantly, the CV (coefficient of variation) of each target bacteria concentration was less than eight, which means that the reproducibility of the proposed fluorescent biosensor is good.

## 4. Conclusions

In this study, we developed a method for the recognition of fluorescent spots based on deep learning via the faster R-CNN algorithm. Furthermore, this approach was used to establish a fluorescent biosensor method for the accurate identification as well as the rapid and sensitive detection of *Salmonella* typhimurium using MNBs, immune FITC FMs and the faster R-CNN algorithm. The experimental results showed that the proposed fluorescent biosensor has the capability to identify fluorescent bacteria, and its minimum limit of detection was found to be as low as 55 CFU/mL. In addition, the average recovery of the biosensor in milk was able to reach 102.74%. At present, the performance of the biosensor is subject to that of the fluorescent microscope and the deep learning algorithm. It is expected that a more automated, accurate, sensitive and rapid means of detection of pathogens can be realized through the use of more portable fluorescence microscope equipment and more advanced deep learning algorithms. Furthermore, the combination of software and hardware achieved via the embedding of deep learning algorithms into smartphone APPs holds promise in terms of achieving image-in-result-out detection of foodborne pathogens. Ultimately, the simultaneous detection of greater numbers of foodborne pathogens, including bacteria and viruses, will be achieved through the use of different antibodies and fluorescent materials of different colors.

## Figures and Tables

**Table 1:** Detection of *Salmonella* typhimurium in milk using this fluorescent biosensor.

No.	Added Bacteria (CFU/mL)	Detected Bacteria (CFU/mL)	Recovery (%)	CV (%)
**1**	69	59	85.31	7.56
**2**	138	152	110.48	6.38
**3**	275	277	100.59	3.86
**4**	550	590	107.33	2.57
**5**	1100	1210	109.99	2.92

## Data Availability

Not applicable.

## Supplementary Materials

The following are available online at https://www.mdpi.com/article/10.3390/bios11110447/s1, Figure S1: (a) Real picture of the cylindrical magnets; (b) 3D printed mold of the sleeve of cylindrical magnets; (c) Real picture of the low-gradient magnetic field equipment; (d) Real picture of the ELISA container; (e) The state of the complexes in an ELISA well before the application of low-gradient magnetic field; (f) The state of the complexes in an ELISA well after the application of low-gradient magnetic field; Figure S2: Hydration particle size analysis of FITC FMs and immune FITC FMs; Figure S3: Situation of training set annotation with LabelImg software; Figure S4: Characterization and identification of fluorescent spots for different concentrations of FITC FMs (a) 50 μg/mL, (b) 10 μg/mL, (c) 5 μg/mL, (d) 1 μg/mL, (e) 0.5 μg/mL, (f) 0.1 μg/mL using the faster R-CNN model. The scale bar is 100 μm; Figure S5: Characterization of magnetic nanobeads (MNBs) and fluorescent complexes under different distributions and different intensities of low-gradient magnetic field. (a) Simulation of magnetic field distribution around a square magnet; (b) The distribution of MNBs and fluorescent complexes on low-gradient magnetic field; Figure S6: The characterization of the size of fluorescent complexes. (a) Morphologies of fluorescent complexes without low-gradient magnetic field; (b) Morphologies of fluorescent complexes with low-gradient magnetic field; (c) The histogram of the diameter of fluorescent complexes on different conditions (with or without low-gradient magnetic field); (d) Characterization of uniform distribution of fluorescent spots under the action of low-gradient magnetic field; Figure S7: Characterization of recognition ability of faster R-CNN algorithm under different exposure times and different intensities of excitation light. (a) The number of fluorescent spots under different exposure times for same field of view; (b) The number of fluorescent spots under different intensities of excitation light for same field of view; (c) Fluorescent images in the same field under different exposure times; (d) Fluorescent images in the same field under different fluorescent intensities, Table S1: Statistical analysis of uniform distribution of fluorescent bacteria under the low-gradient magnetic field; Table S2: Comparison of this proposed fluorescent biosensor with previously reported fluorescent biosensors.
