# Supplementary material for: A Fluorescent Biosensor for Sensitive Detection of Salmonella Typhimurium Using Low-Gradient Magnetic Field and Deep Learning via Faster Region-Based Convolutional Neural Network
Source: Biosensors (Basel). 2021 Nov 11;11(11):447. doi: 10.3390/bios11110447 (PMC8615454; doi:10.3390/bios11110447)
Supplement: Supplementary file 1 [file biosensors-11-00447-s001.zip › biosensors-1426882-supplementary.pdf]

biosensors

Supplementary

A Fluorescent Biosensor for Sensitive Detection of *Salmonella* Typhimurium Using Low-Gradient Magnetic Field and Deep Learning via Faster Region-Based Convolutional Neural Network

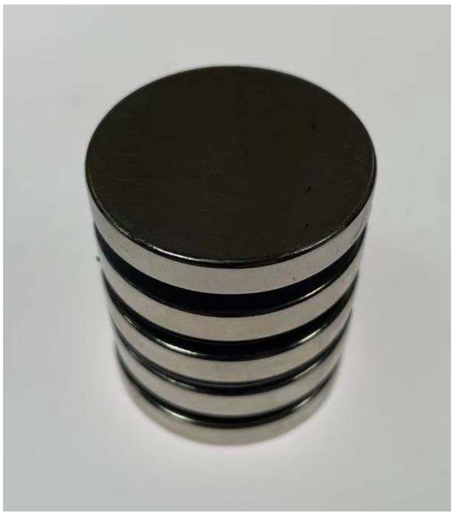

(a)

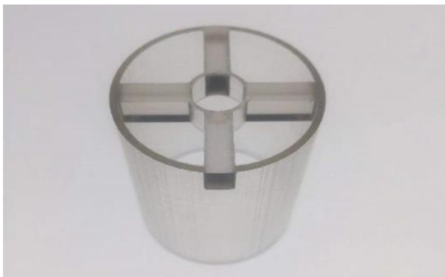

(b)

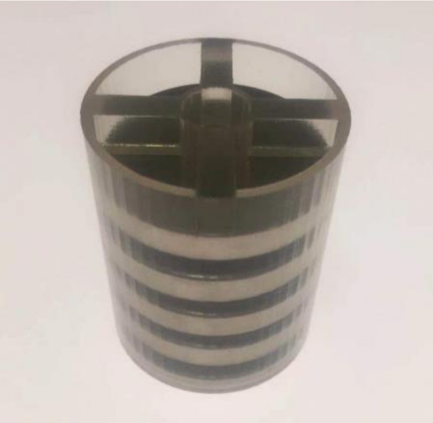

(c)

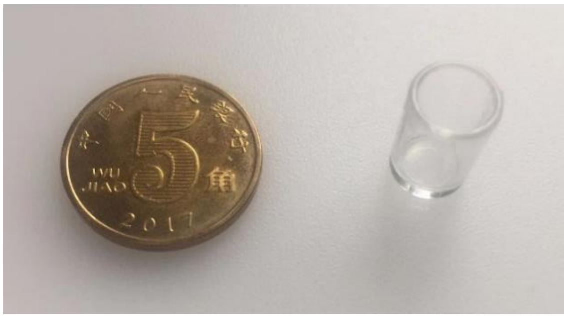

(d)

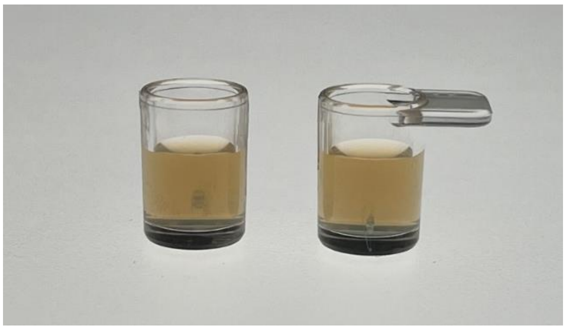

(e)

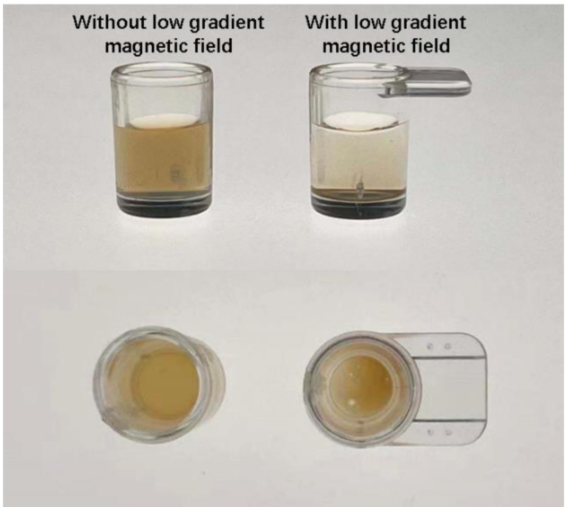

(f)

Figure S1. (a) Real picture of the cylindrical magnets; (b) 3D printed mold of the sleeve of cylindrical magnets; (c) Real picture of the low-gradient magnetic field equipment; (d) Real picture of the ELISA container; (e) The state of the complexes in an ELISA well before the application of low-gradient magnetic field; (f) The state of the complexes in an ELISA well after the application of low-gradient magnetic field.

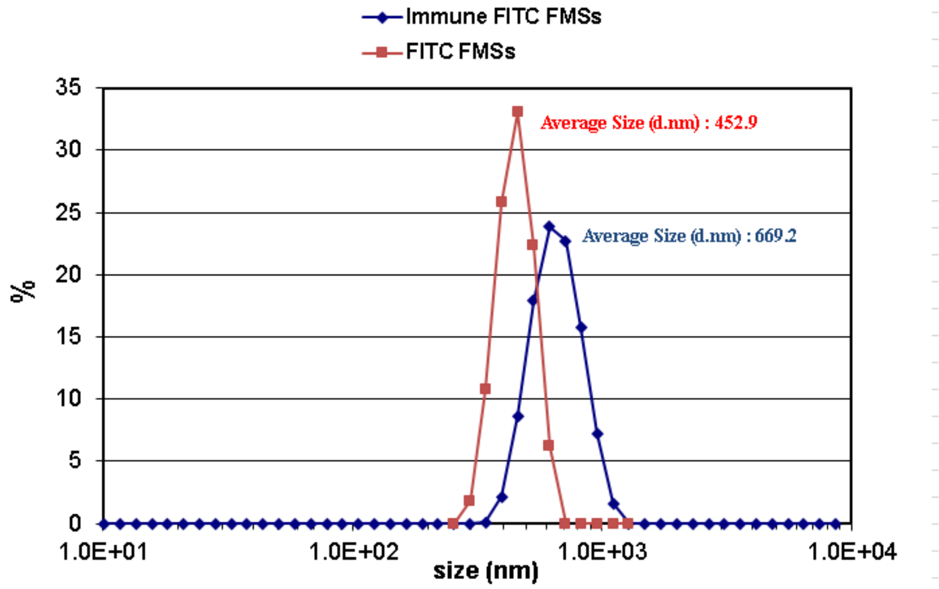

Figure S2. Hydration particle size analysis of FITC FMs and immune FITC FMs.

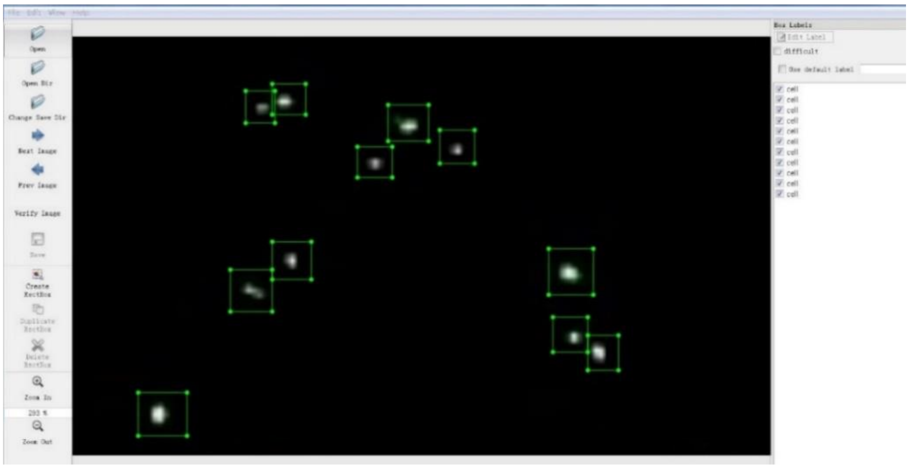

Figure S3. Situation of training set annotation with LabelImg software.

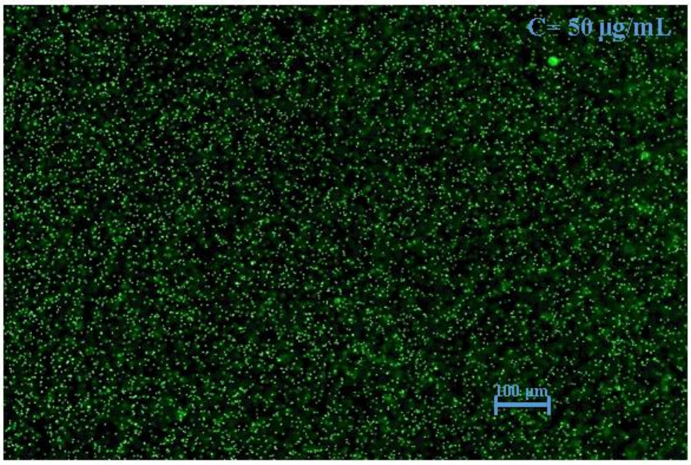

(a)

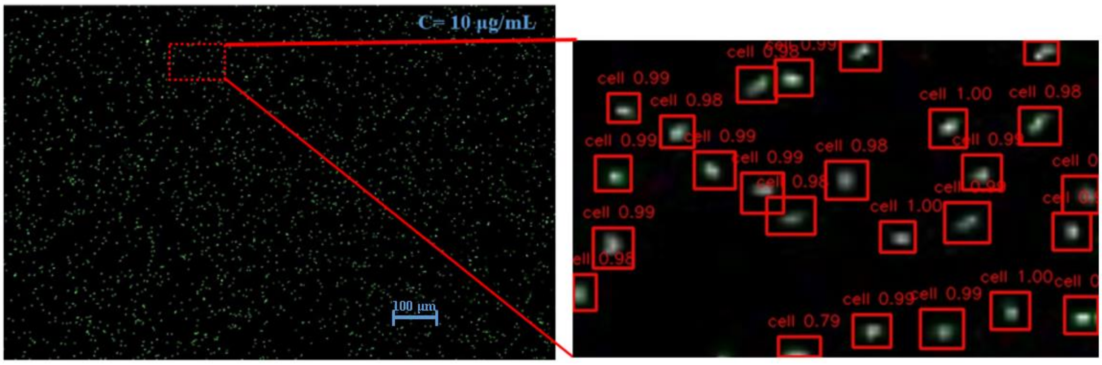

(b)

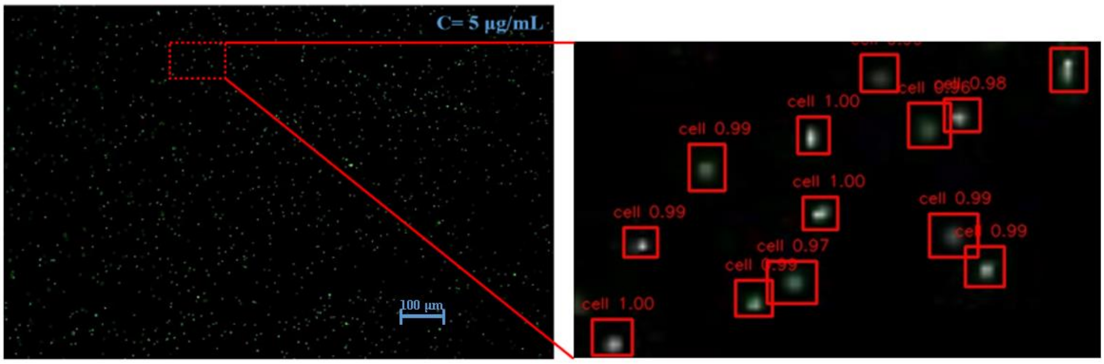

(c)

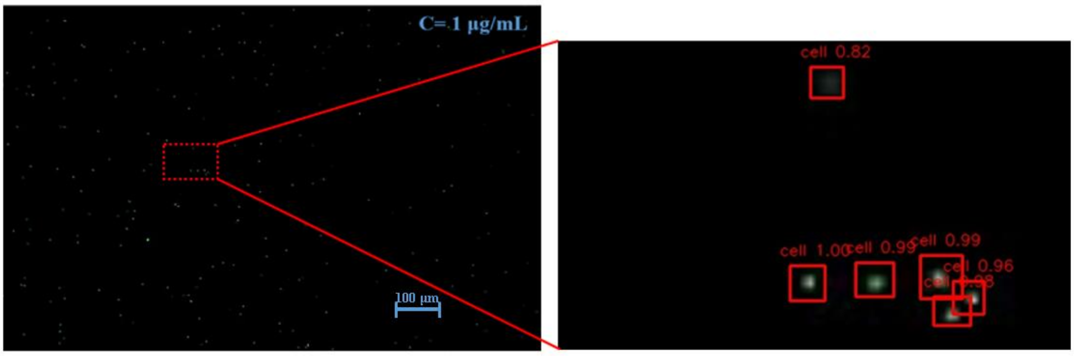

(d)

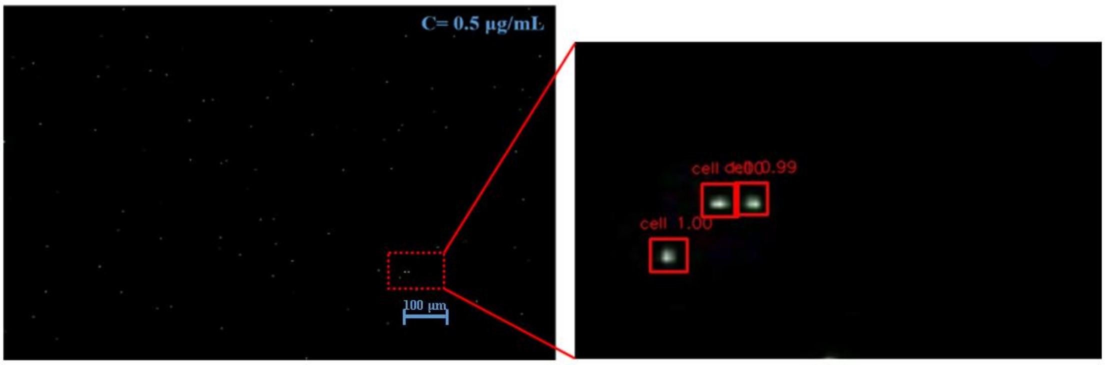

(e)

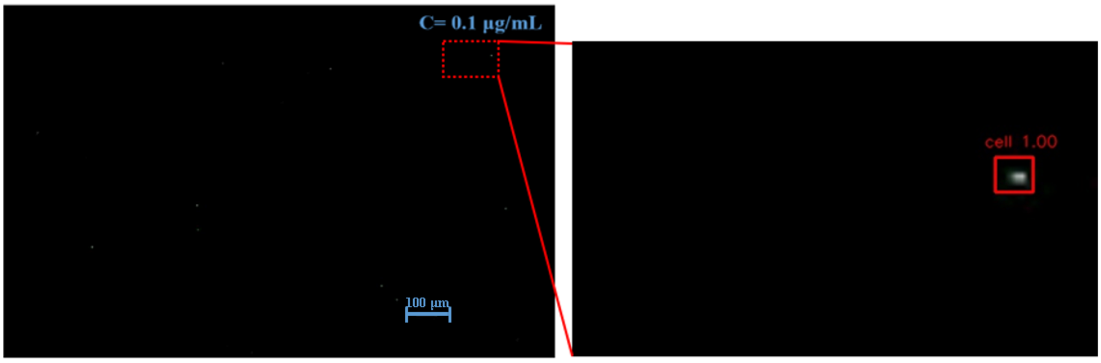

(f)

Figure S4. Characterization and identification of fluorescent spots for different concentrations of FITC FMs (a) 50 μg/mL, (b) 10 μg/mL, (c) 5 μg/mL, (d) 1 μg/mL, (e) 0.5 μg/mL, (f) 0.1 μg/mL using the faster R-CNN model. The scale bar is 100 μm.

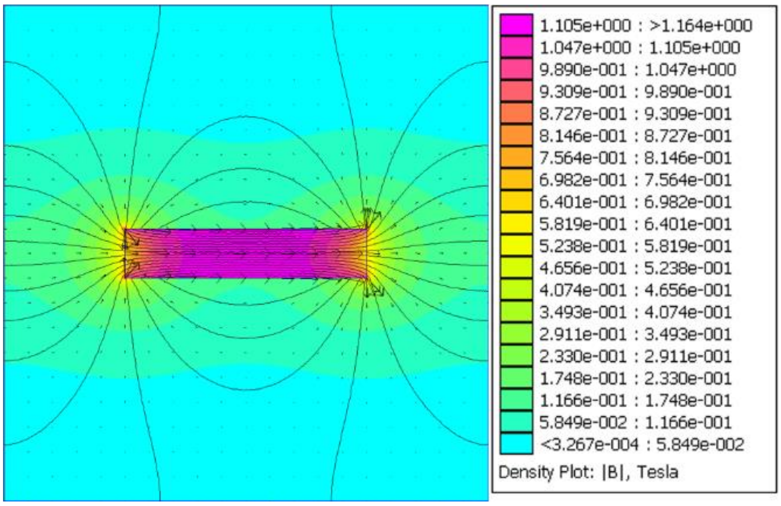

(a)

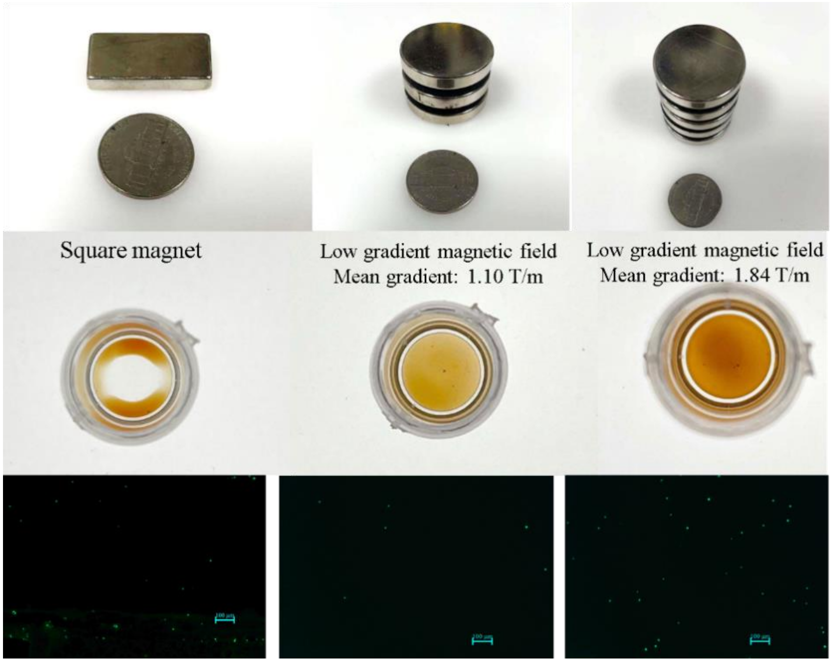

(b)

Figure S5. Characterization of magnetic nanobeads (MNBs) and fluorescent complexes under different distributions and different intensities of low-gradient magnetic field. (a) Simulation of magnetic field distribution around a square magnet; (b) The distribution of MNBs and fluorescent complexes on low-gradient magnetic field.

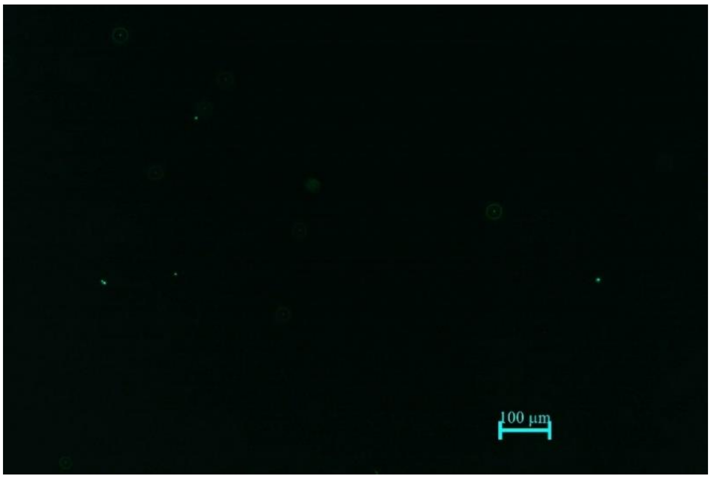

(a)

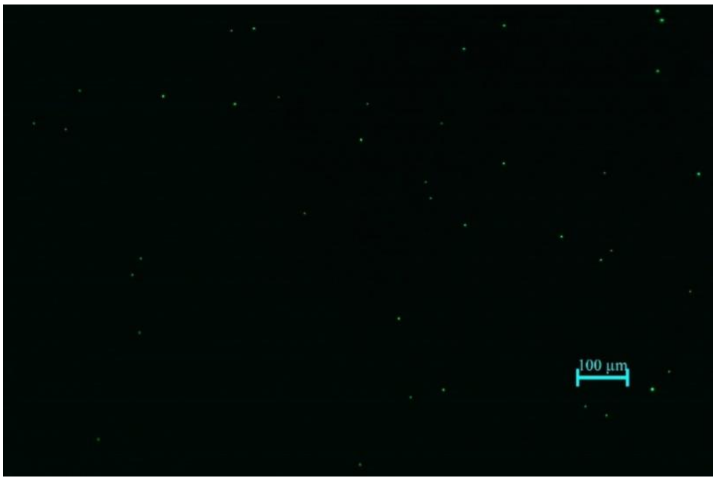

(b)

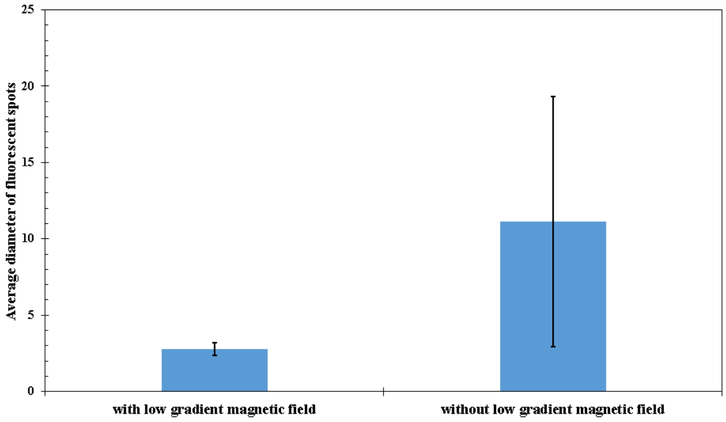

(c)

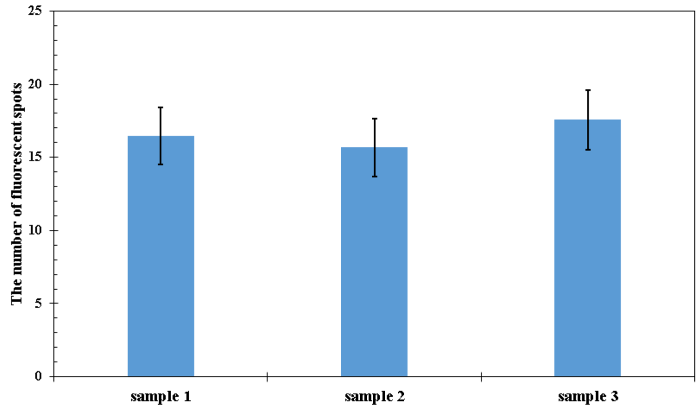

(d)

Figure S6. The characterization of the size of fluorescent complexes. (a) Morphologies of fluorescent complexes without low-gradient magnetic field; (b) Morphologies of fluorescent complexes with low-gradient magnetic field; (c) The histogram of the diameter of fluorescent complexes on different conditions (with or without low-gradient magnetic field); (d) Characterization of uniform distribution of fluorescent spots under the action of low-gradient magnetic field.

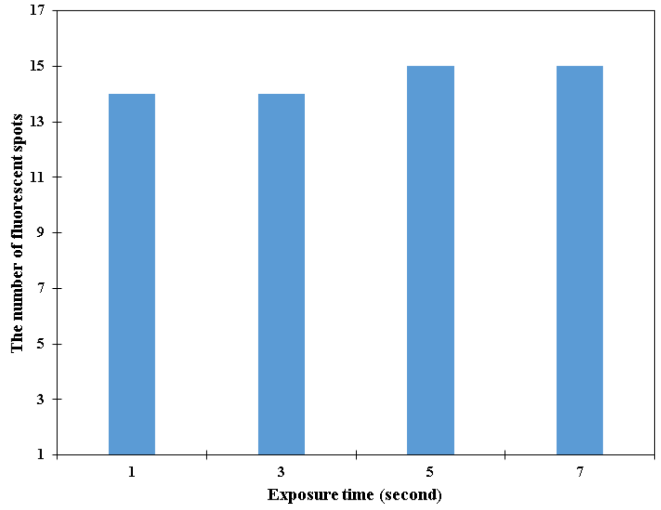

(a)

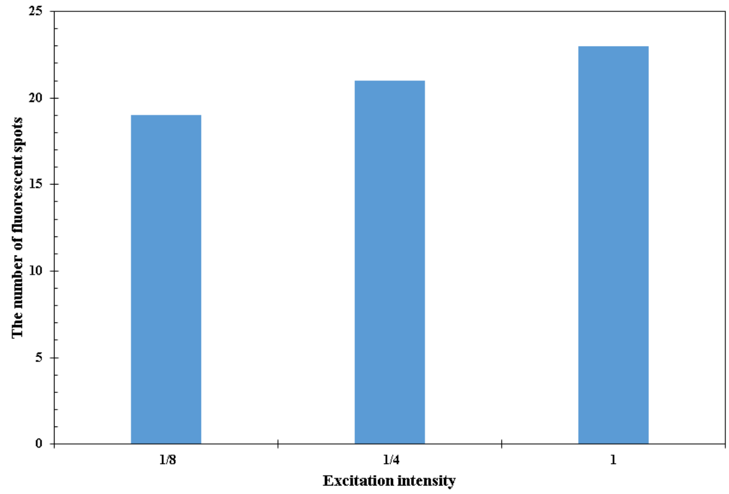

(b)

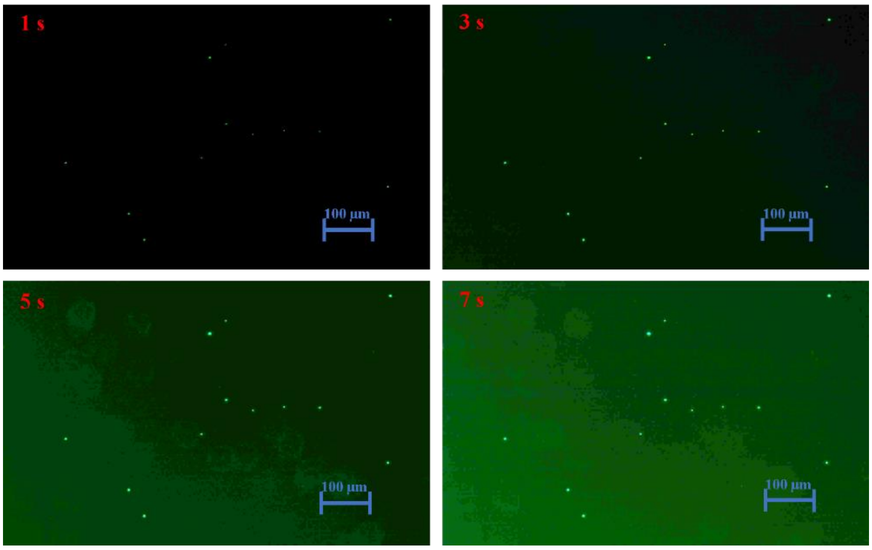

(c)

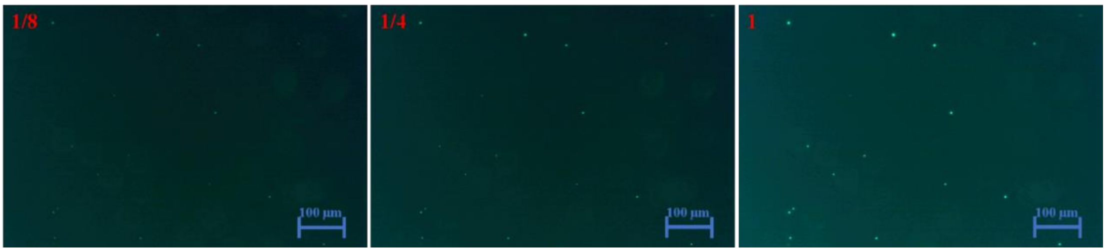

(d)

Figure S7. Characterization of recognition ability of faster R-CNN algorithm under different exposure times and different intensities of excitation light. (a) The number of fluorescent spots under different exposure times for same field of view; (b) The number of fluorescent spots under different intensities of excitation light for same field of view; (c) Fluorescent images in the same field under different exposure times; (d) Fluorescent images in the same field under different fluorescent intensities.

Table S1. Statistical analysis of uniform distribution of fluorescent bacteria under the low-gradient magnetic field.

	**1**	**2**	**3**	**4**	**5**	**6**	**7**	**8**	**9**	**AVERAGE**	**STDEV**	**AVEDEV**	**CV**
**Sample 1**	13	23	16	13	17	17	17	15	17	16.444	2.963	1.951	18.02%
**Sample 2**	15	14	18	14	18	13	14	20	15	15.667	2.398	2.000	15.31%
**Sample 3**	18	17	18	18	17	14	21	22	13	17.556	2.877	2.049	16.39%

*** The mean value from the three measurements.**

Table S2. Comparison of this proposed fluorescent biosensor with previously reported fluorescent biosensors.

**No.**	**Targets**	**Detection limit (CFU/mL)**	**References**
1	*shigella*	1.0×10^4	[1]
2	*Salmonella* typhimurium	1.38×10^2	[2]
3	*Salmonella*	10^2	[3]
4	*Salmonella*	10^2	[4]
5	*E. coli* O157:H7	2.39×10^2	[5]
6	*Salmonella* typhimurium	4.3×10^1	[6]
7	*Salmonella* typhimurium	5.5×10^1	This study

References

[1] F. Zhu, K. He, X. Zhang, D. Wei, S. Wang, Y. Li, Y. Chen, R. Zhao, Study on visual detection of *Shigella* by quantum dot labeling with fluorescence immunoassay strip in meat, *Food Research and Development* 2021, *42 (3)*, 136–140.

[2] Z. Guo, X. Huang, Z. Li, J. Shi, X. Zhai, X. Hu, N. Liang, X. Zou, Rapid and highly sensitive detection of *Salmonella* typhimurium in lettuce by using magnetic fluorescent nanoparticles, *Anal. Methods* 2020, *12(48)*, 5861–5868.

[3] Y. Meng, L. Liu, X. Yang, X. Dai, K. Chen, Rapid detection of *Salmonella* in chicken meat by fluorescent labeled DNA-magnetic graphene oxide magnetic separation technology, *Food and Fermentation Industries* 2020, *46(12)*, 251–257.

[4] W.-W. Cui, L.-L. Xu, Y.-Y. Shi, N. Dong, P. Chen, Rapid Detection of *Salmonella* via Off-on Composite Fluorescent Probe Based on Fluorescence Resonance Energy Transfer, *Chinese Journal of Analytical Chemistry* 2019, *47(12)*, 1973–1980.

[5] Z. Huang, J. Peng, J. Han, G. Zhang, Y. Huang, M. Duan, D. Liu, Y. Xiong, S. Xia, W. Lai, A novel method based on fluorescent magnetic nanobeads for rapid detection of *Escherichia coli* O157:H7, *Food Chemistry* 2019, *276*, 333–341.

[6] L. Hao, L. Xue, F. Huang, G. Cai, W. Qi, M. Zhang, Q.a. Han, Z. Wang, J. Lin, A Microfluidic Biosensor Based on Magnetic Nanoparticle Separation, Quantum Dots Labeling and MnO2 Nanoflower Amplification for Rapid and Sensitive Detection of *Salmonella* Typhimurium, *Micromachines* 2020, *11 (3)*.
